# Evaluation of the *in vitro* antioxidant and antitumor activity of extracts from *Camellia fascicularis* leaves

**DOI:** 10.3389/fchem.2022.1035949

**Published:** 2022-10-31

**Authors:** Xiaowei Peng, Xuhua He, Junrong Tang, Jianying Xiang, Jia Deng, Huan Kan, Yingjun Zhang, Guiliang Zhang, Ping Zhao, Yun Liu

**Affiliations:** ^1^ Key Laboratory of Forest Resources Conservation and Utilization in the Southwest Mountains of China Ministry of Education, Southwest Forestry University, Kunming, China; ^2^ Forest Resources Exploitation and Utilization Engineering Research Center for Grand Health of Yunnan Provincial Universities, Kunming, China; ^3^ State Key Laboratory of Phytochemistry and Plant Resources in West China, Kunming Institute of Botany, Chinese Academy of Sciences, Kunming, China; ^4^ Hekou Management Sub-bureau of Yunnan Daweishan National Nature Reserve Management Bureau, Honghe, China

**Keywords:** *Camellia fascicularis*, extracts, active substances, antioxidant, antitumor

## Abstract

*Camellia fascicularis* is a unique plant rich in bioactive components. However, the isolation of the active substances in *C. fascicularis* leaves *via* sequential extraction with solvents of different polarity and the determination of their antioxidant and antitumor activities have not been reported. In this study, the total methanol extract of *C. fascicularis* leaves was sequentially extracted with different polar solvents, and the corresponding petroleum ether extract (PEE), ethyl acetate extract (EAE), and water extract (WE) were analyzed for their contents in active substances such as flavonoids, polyphenols, polysaccharides, and saponins. The antioxidant ability of the polar extracts was investigated by determining their reducing power and the radical scavenging rate on 1,1-diphenyl-2-picrylhydrazyl (DPPH), 2,2′-azino-bis (3-ethylbenzothiazoline-6-sulfonic acid) (ABTS), and hydroxyl radicals, and CCK-8 and Annexin-FITC/propidium iodide staining assays were conducted to investigate their inhibitory effects on HCCLM6 and HGC27 tumor cells. The results showed that PEE had a high saponin content of 197.35 ± 16.21 mg OAE/g, while EAE and WE exhibited a relatively higher polysaccharide content of 254.37 ± 1.99 and 373.27 ± 8.67 mg GE/g, respectively. The EAE demonstrated the greatest reducing power and the strongest clearing abilities on ABTS and DPPH radicals with respective EC_50_ values of 343.45 ± 20.12 and 14.07 ± 0.06 μg/ml. Moreover, the antitumor ability of the different polar extracts was dose-dependent, with WE showing the most potent inhibitory ability against HCCLM6 and HGC27 cells.

## 1 Introduction

*Camellia fascicularis*, a genus of *Camellia* in the family Theaceae, is an endemic plant in Yunnan province, China. *C. fascicularis*, which is a rare species resource with unique golden petals also known as “the giant panda in the plant kingdom,” “the queen of the tea family,” and “the living fossil of plants,” was first discovered in Hekou County and only distributed in Gejiu, Maguan, and Hekou counties (Liu et al., 2018). *C. fascicularis* leaves (CFLs) possess high amino acid and mineral content and are considered as an edible plant resource with high nutritional and health values (Hu et al., 2021). Bioactive components such as polyphenols, flavonoids, and saponins with strong *in vitro* antioxidant activity were also found in CFLs (Liu et al., 2019). In addition, polyphenols from CFLs were reported to exert anti-inflammatory properties *via* suppressing NF-KB and MAPK signaling pathways (Gao et al., 2022). However, to the best of our knowledge, the isolation of the active substances in CFLs according to the polarity of the extraction solvent and their antioxidant and antitumor activities have not been reported.

Extraction and isolation of natural drugs are one of the main research goals of natural medicinal chemistry (Takahashi et al., 2014). Natural drugs with clinically proven efficacy or having biological activity identified *via* activity screening must be first extracted and isolated to obtain the corresponding active substances. In the extraction process, bioactive components with different polarities can be extracted following the similarity compatibility principle and used as targets to determine the suitability of the extraction solvent (Abarca-Vargas et al., 2016). The extraction of functional components from plant resources using different polar solvents, among which the most commonly used are water, acetonitrile, methanol, ethanol, ethyl acetate, and petroleum ether has been extensively investigated. For example, Chuen et al. (2016) investigated the impact of water, methanol, ethanol, acetone extractions on the total phenolic content of *Davidsonia pruriens*, and Chang et al. (2021) explored the differences in the nutrient composition of *Sechium edule* shoot extracts obtained separately by water, hexane, methanol, and ethyl acetate.

The main objectives of this investigation were to preliminarily extract the active substances of CFLs and to investigate their antioxidant and antitumor properties. The total methanol extract of CFLs was subjected to sequential extraction with solvents of different polarity, i.e., petroleum ether, ethyl acetate, and water, and the corresponding extracts were analyzed to determine their respective contents in active substances such as flavonoids, polyphenols, polysaccharides, and saponins. Moreover, the antioxidant and antitumor activities of the petroleum ether extract (PEE), ethyl acetate extract (EAE), and water extract (WE) were further compared by conducting 1,1-diphenyl-2-picrylhydrazyl (DPPH), 2,2′-azino-bis (3-ethylbenzothiazoline-6-sulfonic acid) (ABTS), and hydroxyl radical assays and reducing power and cell toxicity analyses. The present study will provide an experimental basis for the isolation and screening of active substances with antioxidant and antitumor effects from CFLs, as well as the production of natural pharmaceuticals*.*

## 2 Materials and methods

### 2.1 Plant material

The voucher specimen (52860) of *Camellia fascicularis* identified by taxonomist Min Tianlu was stored at herbarium of Kunming Institute of Botany, Chinese Academy of Sciences. CFLs were obtained in Dawei Mountain (103.95 N, 22.66 E) in Hekou County, Yunnan Province, China in December 2019, which was identified as *Camellia fascicularis* by Professor Xiang Jianying, a taxonomist at Southwest Forestry University. CFLs were hot-air dried at 45°C until constant weight, pulverized to powder, then screened with 60-mesh sieves. The powders were kept at 4°C for further analysis.

### 2.2 Chemicals and reagents

DPPH and ABTS were acquired from Shanghai Yuanye Bio-Technology Co., Ltd. (Shanghai, China). Cell Counting Kit-8 (CCK-8) was acquired from Shenyang Wanlei Bio-Technology Co., Ltd. (Shenyang, China). All other chemicals were analytical grade.

### 2.3 Preparation of *C. fascicularis* leaves extracts

**FIGURE 1:**
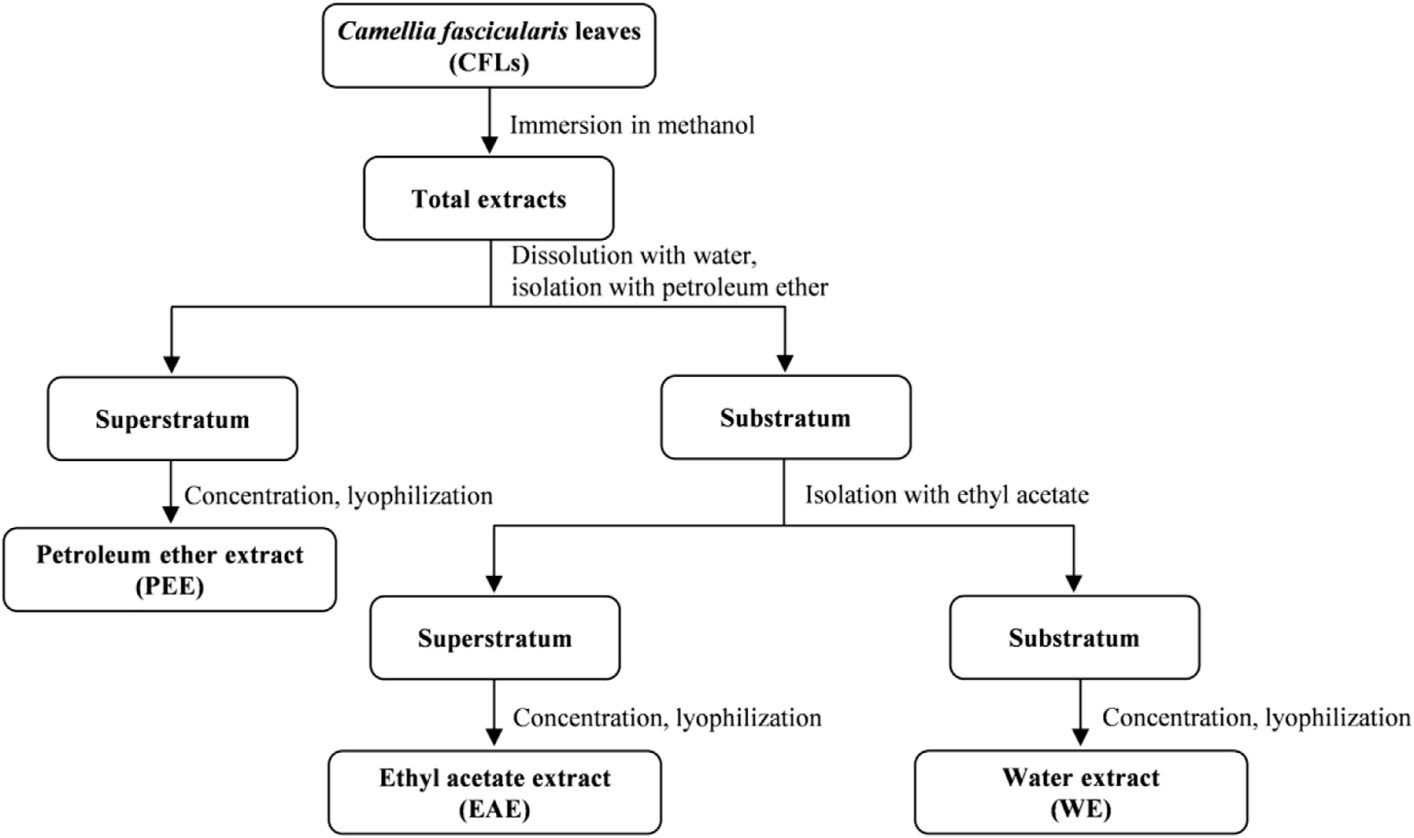
Extraction process of CFLs.

The extraction procedures of CFLs were schematically depicted in Figure 1. First, 10.68 kg of CFLs was immersed in methanol at room temperature, and the total extracts were obtained by filtering, concentrating, and drying under negative pressure. Then, the total extract was fully dissolved in distilled water under ultrasonic action and isolated sequentially with equal amounts of petroleum ether and ethyl acetate. Finally, 122.0 g of PEE, 177.8 g of EAE, and 115.8 g of WE were obtained after concentration and freeze-drying (Hu et al., 2017).

### 2.4 Determination of the content in active substances

#### 2.4.1 Total flavonoid content

The TFC of CFLs extracts was measured by Liao’s approach with slight modifications (Liao et al., 2021). Briefly, the extracts were reacted with NaNO_2_, Al(NO_3_)_3_, and NaOH in turn, and the absorption value of the mixtures was then determined using an ultraviolet–visible spectrophotometer (UV-2600, Shimadzu, Japan). A calibration curve (y = 7.5357x + 0.0117, *R* ^2^ = 0.9999) was constructed using a rutin standard, and the TFC was defined by rutin equivalent (RE; mg) per dry weight of extracts (g).

#### 2.4.2 Total polyphenol content

The TPC of CFLs extracts was measured according to Emanet’s method with slight variations (Emanet et al., 2022). Briefly, the extracts were reacted with Folin–Ciocalteu reagent and Na_2_CO_3_ in turn, and the absorption value of the mixtures was then measured. A calibration curve (y = 82.3714x + 0.0392, *R* ^2^ = 0.9998) was constructed with gallic acid as standard, and the TPC was defined by gallic acid equivalent (GAE; mg) per dry weight of extracts (g).

#### 2.4.3 Polysaccharide content

The PC of the CFLs extracts was determined using Wang’s method with minor modifications (Wang et al., 2018). First, the extracts were refluxed in 95% ethanol by 6 h for the removal of oligosaccharides and monosaccharides. After that, 1 ml residue solution was reacted with 1 ml of 5% phenol solution and 5 ml concentrated sulfuric acid in turn, and the absorption value of the mixtures was determined at 490 nm. A calibration curve (y = 9.5961x + 0.00002, *R* ^2^ = 0.9996) was obtained using a glucose standard, and the PC was defined as glucose equivalent (GE; mg) per dry weight of extracts (g).

#### 2.4.4 Total saponin content

Akbari’s method with slight modifications (Akbari et al., 2019) was used to determine the TSC of the CFLs extracts. Briefly, the extracts were reacted with a vanillin–acetic acid solution, perchloric acid, and acetic acid in turn, and the absorption value of the mixtures was then measured. An oleanolic acid standard was used to obtain a calibration curve (y = 7.5895x − 0.0084, *R* ^2^ = 0.9993), and the TSC was defined as oleanolic acid equivalent (OAE; mg) per dry weight of extracts (g).

### 2.5 *In vitro* antioxidant activity

#### 2.5.1 DPPH assay

A literature procedure slightly modified was employed for evaluating the clearing effect of CFLs extracts on DPPH radical (Zhu et al., 2022). Specifically, 0.2 ml of CFLs extracts in varying concentrations (10–60 μg/ml) was combined into 0.2 ml of a DPPH radical ethanol solution. The mixed solutions were then stored for 30 min at 37°C, then the absorption value at 517 nm was detected using a microplate reader (SpectraMax190, Molecular Devices, United States).

#### 2.5.2 ABTS assay

Swat’s method with minor modifications was applied to evaluate the clearing ability of CFLs extracts on ABTS radicals (Swat et al., 2019). Briefly, 0.02 ml of CFLs extracts in varying concentrations (300–1300 μg/ml) was combined into 0.38 ml of an ABTS radical cation solution. The mixed solutions were stored for 5 min, and the absorption value at 734 nm was detected.

#### 2.5.3 Hydroxyl assay

Mao’s method with minor modifications was employed for investigating the clearing effect of CFLs extracts on hydroxyl radical (Mao et al., 2013). In brief, 0.2 ml of CFLs extracts in varying concentrations (1000–11000 μg/ml) was reacted with an FeSO_4_ aqueous solution, a salicylic acid ethanol solution, and H_2_O_2_ in turn, and the absorption value of the mixtures at 510 nm was then measured.

#### 2.5.4 Reducing power assay

Bao’s method with minor variations was adopted to study the reducing power of the CFLs extracts (Bao et al., 2020). Specifically, 0.1 ml of CFLs extracts in varying concentrations (300–1300 μg/ml) was reacted with phosphate buffer, potassium ferricyanide, and trichloroacetic acid in turn, and the absorption value of the mixtures at 700 nm was then determined.

### 2.6 *In vitro* antitumor activity

#### 2.6.1 Cell culture

Human hepatocellular carcinoma HCCLM6 and Human gastric cancer cell line HGC27 cells were obtained from Kunming Cell Bank (CAS, China) and preserved in Dulbecco’s modified Eagle medium (Gibco, United States). The media was added in penicillin (100 μg/ml) and 10% (v/v) fetal bovine serum at 37°C in an incubator (BB15, Thermo, United States) with a supply of 5% CO_2_ (Wang et al., 2019).

#### 2.6.2 Cell viability

To evaluate the cytotoxicity of the CFLs extracts against HCCLM6 and HGC27 cells, the cell viability was quantified using CCK-8 (Zhang et al., 2020). In a 96-well microplate, cells were plated with 1 × 10^5^/ml density and cultured for 24 h at 37°C. The cells were treated with 10 μL different concentrations of CFLs extracts DMSO solution (0, 50, 100, 150, 200, and 250 μg/ml) for 24 h. Then, each well was mixed with 10 µL CCK-8 solution and incubation was maintained for 1 h. The absorption value of the solution in well was determined using a microplate reader at 450 nm.

#### 2.6.3 Cell apoptosis

The cell apoptosis was detected following Dai’s method with minor modifications (Dai et al., 2021). Briefly, HCCLM6 and HGC27 cells in log growth phase were transferred to 6-well plates at a density of 1 × 10^5^/ml and incubated at 37°C for 24 h in 5% CO_2_. Then, CFLs extracts in different concentrations (0, 50, 150 and 250 μg/ml) was applied and incubation was continued for 24 h. After digestion, centrifugation, and cleaning twice, the cells were stained for 15 min in the dark with Annexin V-FITC (5 μL) and propidium iodide (PI) (5 μL). A flow cytometry (FACSCanto II, BD, United States) was performed to identify the apoptotic cells.

### 2.7 Statistical analysis

Origin 2018 and GraphPad Prism eight were used for mapping, and all measurements were performed in triplicates. Statistical differences among groups were performed using Student’s t-test.

## 3 Result

### 3.1 Active substances of *C. fascicularis* extracts

As shown in Table 1, TFC, TPC, PC, and TSC differed significantly between the different polar extracts (*p* < 0.05). Thus, TFC in three extracts ranged from 91.24 ± 13.14 to 115.38 ± 6.86 mg RE/g, with the content being significantly higher in WE and EAE than in PEE. TPC in the three extracts was significantly different, EAE had the highest TPC of 164.18 ± 12.05 mg GAE/g, WE possessed the TPC of 132.77 ± 5.81 mg GAE/g and PEE had the lowest TPC of 55.48 ± 5.02 mg GAE/g. Meanwhile, PC was the highest in WE followed by EAE and PEE with 373.27 ± 8.67, 254.37 ± 1.99, and 134.42 ± 2.44 mg GE/g, respectively. In contrast, TSC was the lowest in EAE at 100.95 ± 12.39 mg OAE/g and the highest in PEE at 197.35 ± 16.21 mg OAE/g.

**TABLE 1:** Active substances of CFLs extracts.

Extracts	TFC (mg RE/g)	TPC (mg GAE/g)	PC (mg GE/g)	TSC (mg OAE/g)
PEE	91.24 ± 13.14^c^	55.48 ± 5.02^c^	134.42 ± 2.44^c^	197.35 ± 16.21^a^
EAE	100.59 ± 13.04^ab^	164.18 ± 12.05^a^	254.37 ± 1.99^b^	100.95 ± 12.39^c^
WE	115.38 ± 6.86^a^	132.77 ± 5.81^b^	373.27 ± 8.67^a^	168.67 ± 6.44^b^

Letters (a–c) indicate statistically significant differences between CFLs, extracts (*p* < 0.05).

### 3.2 *In vitro* antioxidant activity of *C. fascicularis* extracts

The antioxidant properties of plant resources have become one of the most important indicators for evaluating their potential use in medicine (Li et al., 2020). In this work, the antioxidant capacities of the three CFLs extracts were determined according to DPPH, ABTS, hydroxyl radicals and reducing power assays using vitamin C (Vc) as a positive control.

#### 3.2.1 DPPH radical clearing capacity

**FIGURE 2:**
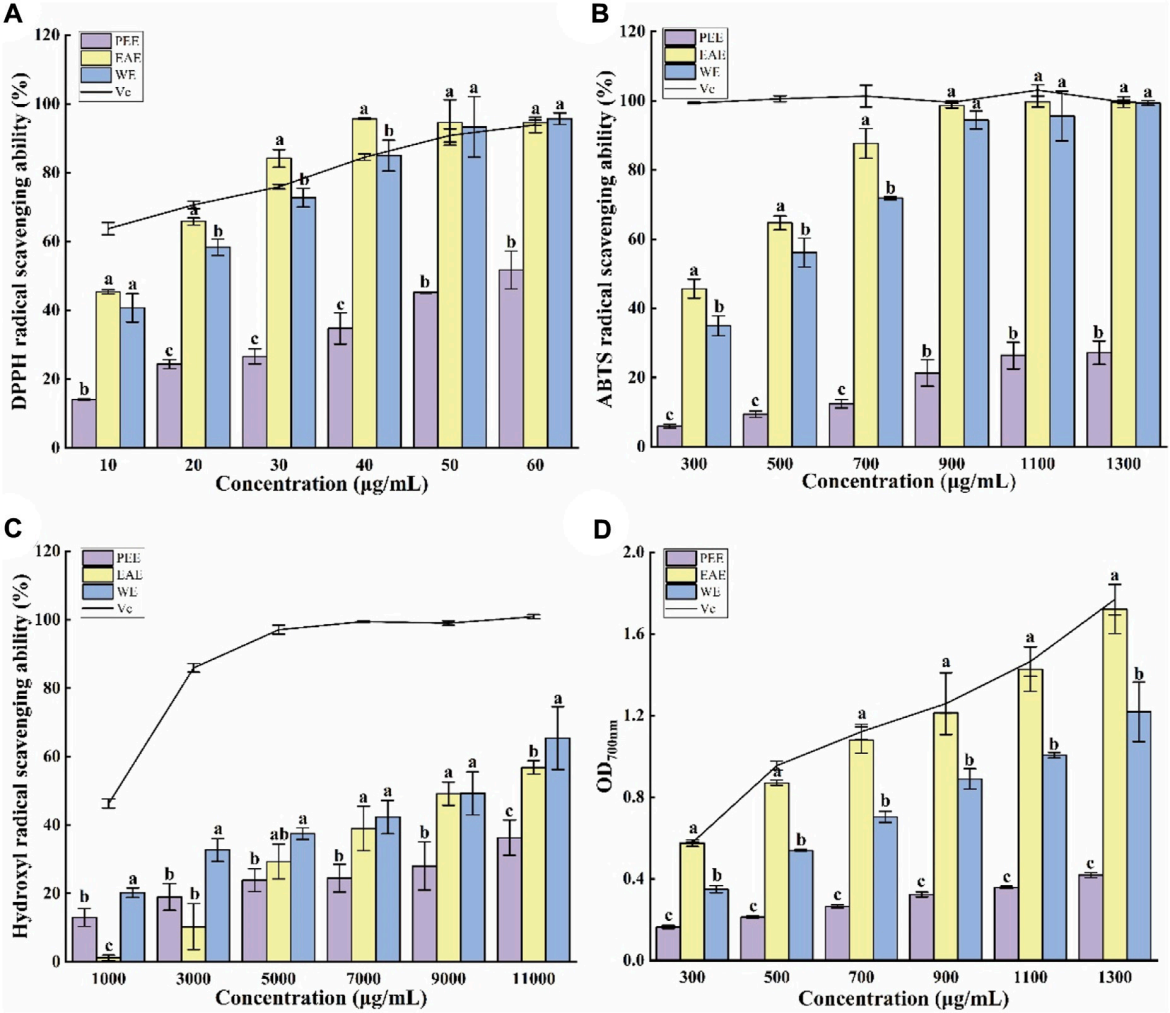
Antioxidant capacities of CFLs extracts. **(A)** DPPH radical scavenging ability, **(B)** ABTS radical scavenging ability, **(C)** hydroxyl radical scavenging ability, and **(D)** reducing power. Letters (a‐c) indicate statistically significant differences between the extracts (*p* < 0.05).

In ethanol solution, DPPH radicals have a high absorption peak at 517 nm. Mixing of a DPPH ethanol solution with antioxidants results in a color lightening, and the fading degree is positively correlated with the ability of the antioxidants to scavenge free radicals (Wang et al., 2015). As shown in Figure 2A, the three CFLs extracts exhibited a certain ability to scavenge DPPH radicals. The clearance of three extracts improved as concentration (10–60 μg/ml) increased, where EAE exceeded Vc at 30–60 μg/ml and WE was close to Vc at 40–60 μg/ml, indicating that EAE and WE possessed efficient DPPH radical clearing capacity. The clearance of PEE was significantly weaker than that of EAE and WE (*p* < 0.05), which may be due to its lower content in flavonoids, polyphenols, and polysaccharides. The 50% maximal effect concentration (EC_50_) of DPPH radical scavenging by PEE, EAE, and WE was 57.68 ± 4.50, 14.07 ± 0.06, and 14.76 ± 3.78 μg/ml, respectively. In general, EAE exhibited the highest potential for scavenging DPPH radicals, followed by WE and PEE. This is consistent with the results of Hu’s DPPH assay on *Astragalus chinensis* (Hu et al., 2017).

#### 3.2.2 ABTS radical clearing capacity

The blue-green color of an ABTS radical solution becomes lighter after reacting with antioxidants, with a more intense lightening indicating a stronger antioxidant capacity (Luo et al., 2010). Figure 2B shows the scavenging effect of three CFLs extracts on ABTS radicals. All three extracts demonstrated strong scavenging ability at 300–1300 μg/ml with dose-dependent effects. The ABTS scavenging rate of EAE and WE was significantly higher than that of PEE, with respective EC_50_ values of 343.45 ± 20.12, 432.72 ± 12.33 and, 2354.92 ± 288.03 μg/ml.

#### 3.2.3 Hydroxyl radical clearing capacity

Hydroxyl radicals are active and harmful species that can induce aging and several diseases in the human body. Therefore, their elimination is extremely significant for human health (Su and Li, 2019). As shown in Figure 2C, WE exhibited the strongest scavenging capacity for hydroxyl radicals among the three CFLs extracts, which contrasts with the findings of the DPPH and ABTS radical scavenging tests. This may be due to the high PC in WE. The EC_50_ values of the hydroxyl radical scavenging by PEE, EAE, and WE were 15294.24 ± 88.14, 9262.31 ± 235.76, and 7913.80 ± 89.55 μg/ml, respectively. Similarly, it was also reported that the hydroxyl radical scavenging ability of EAE from *Andrographis paniculata* was higher than that of PEE (Saranya et al., 2010).

#### 3.2.4 Reducing power

The reducing power intimately associated with the antioxidant capacity and can be used as an indication of the latter, and high absorbance represented strong reducing power (Liu et al., 2014). As shown in Figure 2D, the absorption value of the mixtures increased with the concentration, suggesting that the antioxidant capacity also increased gradually. The absorption value of EAE at 1300 μg/ml was 1.72 ± 0.12, which was close to the values of 1.77 ± 0.08 obtained for Vc and significantly higher than the value of 1.22 ± 0.15 for WE and 0.42 ± 0.01 for PEE (*p* < 0.05). Hu et al. (2017) also found that the reducing power of EAE from *Astragalus chinensis* was higher than that of WE and PEE.

### 3.3 *In vitro* antitumor activity of *C. fascicularis* extracts

#### 3.3.1 Effects of *C. fascicularis* extracts on HCCLM6 and HGC27 cell activity

**FIGURE 3:**
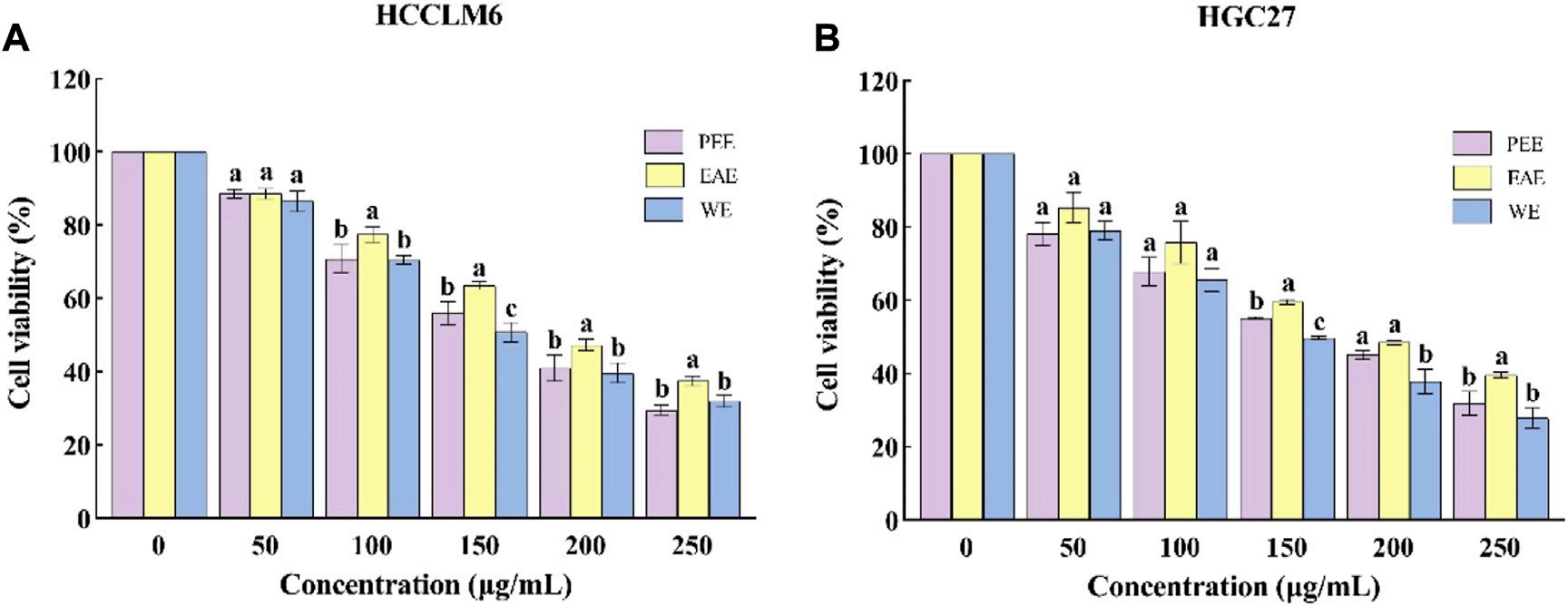
Cytotoxic activity of CFLs extracts. **(A)** Cytotoxic activity of CFLs extracts on HCCLM6 cells and **(B)** cytotoxic activity of CFLs extracts on HGC27 cells. Letters (a–c) represent statistically significant differences between the extracts (*p* < 0.05).

To investigate the antitumor activity of CFL extracts, the cytotoxic effects of the three extracts were assessed on two different cell lines, i.e., HCCLM6 and HGC27cells. As shown in Figure 3A, the three extracts significantly inhibited (*p* < 0.05) the growth of HCCLM6 cells with dose-dependent properties. The viability of HCCLM6 cells treated with PEE, EAE, and WE was below 50% (41.07 ± 3.60%, 47.39 ± 1.60%, and 39.76 ± 2.72%, respectively) at 200 μg/ml. The EC_50_ values of PEE, EAE, and WE were respective 167.87 ± 6.25, 205.09 ± 3.48, and 162.12 ± 5.88 μg/ml. Zhang et al. (2012) have found that the EC_50_ of *Celastrus orbiculatus* extracts against HCCLM6 cell was 282.71 μg/ml, implying that PEE, EAE and WE possessed higher inhibitory effect on HCCLM6 cell than *Celastrus orbiculatus* extracts. As shown in Figure 3B, the CFLs extracts also exerted a significant inhibitory effect on HGC27 cells (*p* < 0.05). At 250 μg/ml, the viability of HGC27 cells treated with PEE, EAE, and WE was 31.96 ± 3.26%, 39.67 ± 0.78%, and 27.92 ± 2.79%, respectively. The respective EC_50_ values of PEE, EAE, and WE were 167.44 ± 8.09, 200.76 ± 11.34, and 147.42 ± 12.42 μg/ml, which were higher than the EC_50_ values of betulonic acid (34.13 μg/ml) against HGC27 cell (Chu et al., 2022). Notably, WE showed the smallest EC_50_ against HCCLM6 and HGC27 cells, indicating its strongest inhibitory activity. Overall, the CFLs extracts displayed significant toxic effects on HCCLM6 and HGC27 cells, among which the inhibitory effect of WE was the strongest. This is probably due to the fact that WE was richer in polysaccharides with certain antitumor effects. Antitumor activity has been reported as one of the typical properties of plant polysaccharides (Dong et al., 2016).

#### 3.3.2 Effects of *C. fascicularis* extracts on HCCLM6 and HGC27 cell apoptosis

**FIGURE 4:**
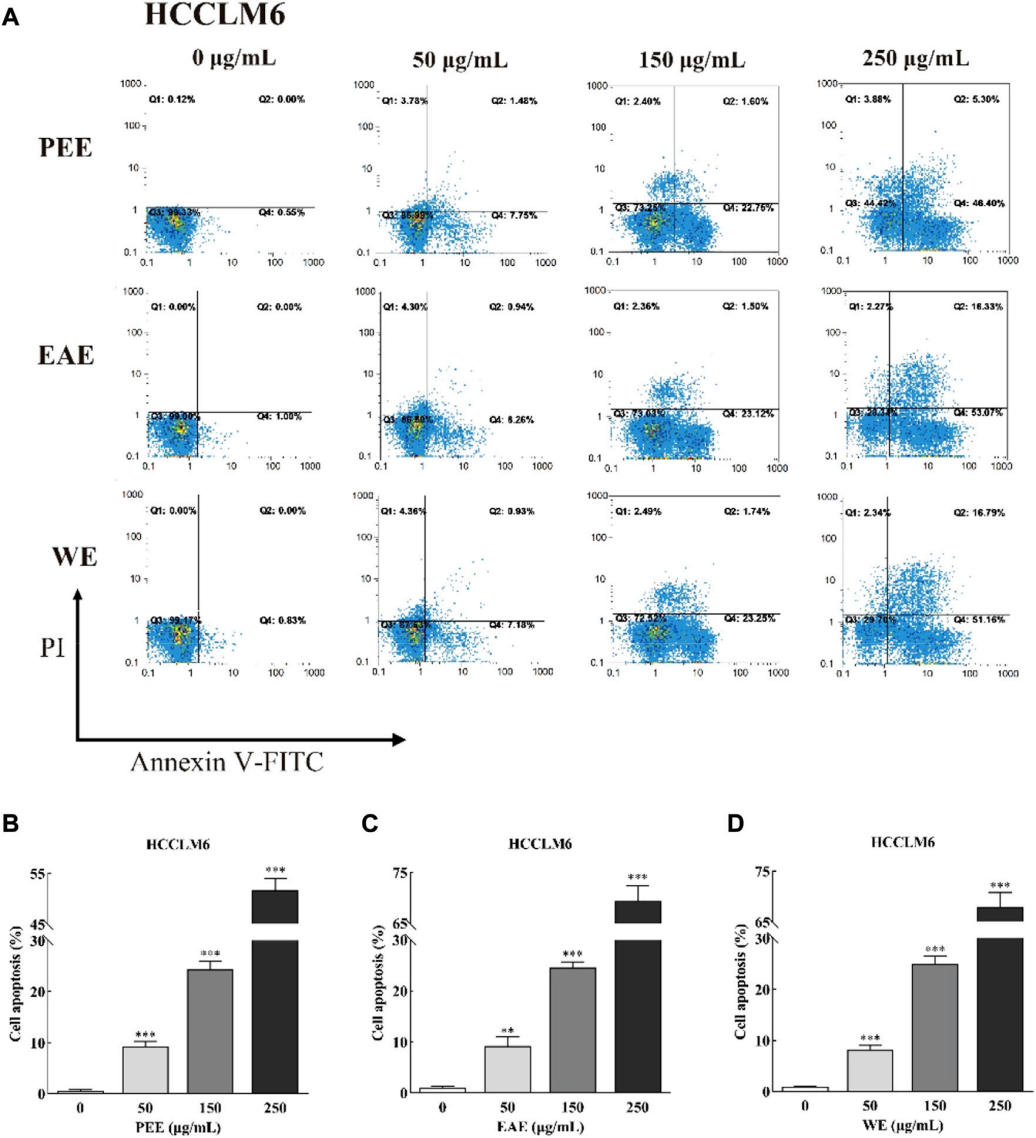
**(A)** Flow cytometry plots and **(B–D)** early and late cell apoptosis rate of HCCLM6 treated with CFLs extracts. **, *p* < 0.01; ***, *p* < 0.001 compared with the control group.

**FIGURE 5:**
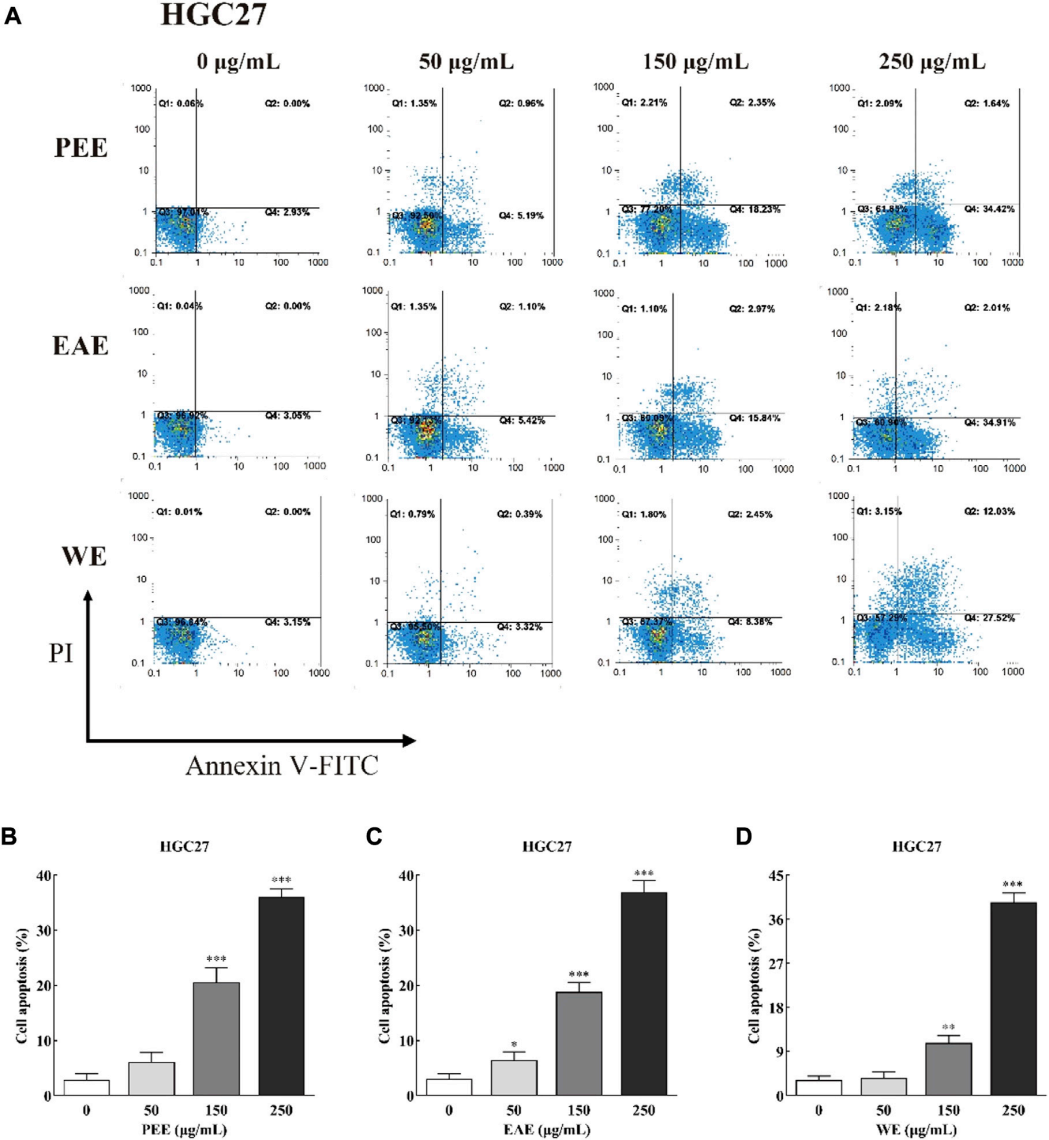
**(A)** Flow cytometry plots and **(B–D)** early and late apoptosis rate of HGC27 treated with CFLs extracts. *, *p* < 0.05; **, *p* < 0.01; ***, *p* < 0.001 compared with the control group.

Exposure of phosphatidylserine to the cell surface is the early feature of apoptosis, which can be identified with annexin V labeled by the fluorescent dye FITC. After the membrane rupture, necrotic cells also bind to annexin V. Thus, cells can be simultaneously labeled with PI (Henry et al., 2013). In the top–right quadrant (Q2) of the flow cytometry plots shown in Figures 4, 5, late apoptotic cells were positive with PI and Annexin V-FITC. Meanwhile, in the bottom–right quadrant (Q4), early apoptotic cells were positive for Annexin V-FITC and negative for PI. As shown in the figures, treatment of HCCLM6 and HGC27 cells with increasing concentrations of PEE, EAE, and WE resulted in enhanced Annexin V-FITC staining, indicating an increase in early apoptotic cells. As shown in Figures 4B–D and Figures 5B–D, the percentages of apoptotic HCCLM6 cells treated with PEE, EAE, and WE were 51.70 ± 2.31%, 69.40 ± 3.05%, and 67.95 ± 2.85% at 250 μg/ml, respectively, and the percentages of apoptotic HGC27 cells were 36.06 ± 1.47%, 36.92 ± 2.13%, and 37.55 ± 1.85%, respectively. These findings suggested that the toxic effect of CFLs extracts on HCCLM6 cells was higher than on HGC27 cells and exhibited a dose-dependent relationship, furthermore, their antitumor effects were mainly achieved by promoting early apoptosis of cancer cells.

### 3.4 Correlation analysis

**FIGURE 6:**
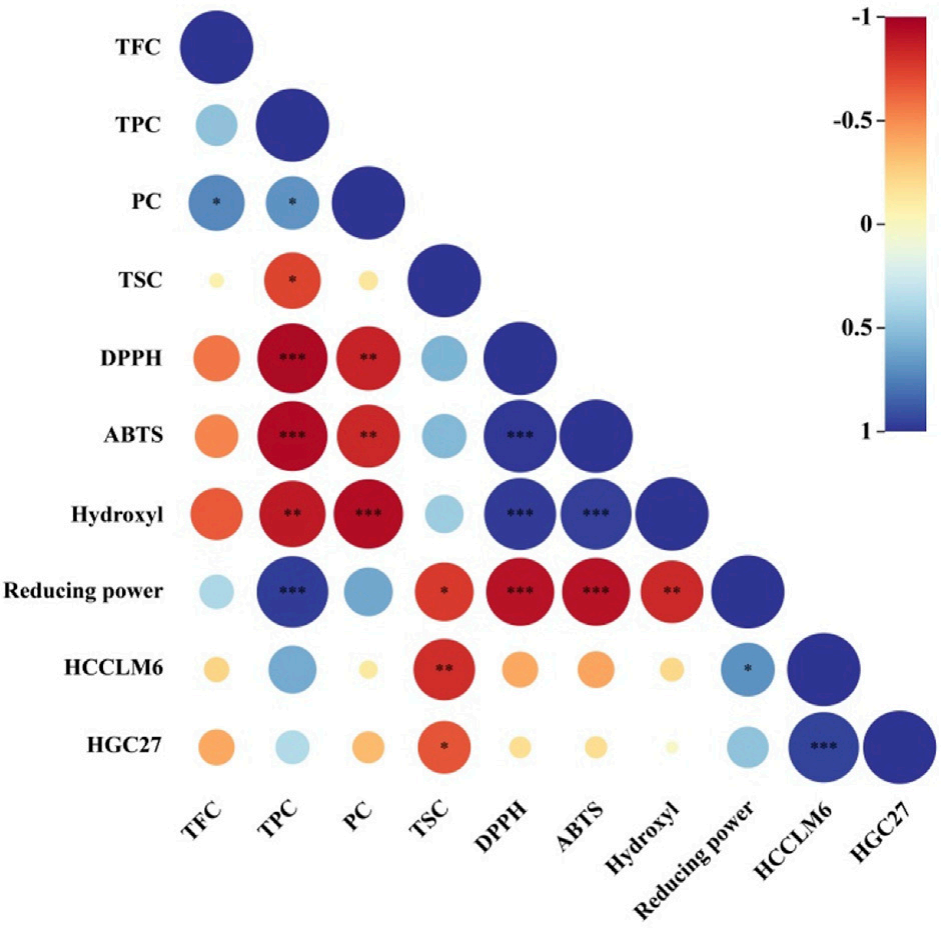
Heat map of Pearson’s correlation coefficient between active substances and *in vitro* antioxidant and antitumor activity. * Significant (*p* < 0.05), ** highly significant (*p* < 0.01), *** extremely significant (*p* < 0.001).

The relationship between the active substances and *in vitro* antioxidant and antitumor activities was assessed using Pearson’s correlation coefficients. As shown in Figure 6, the EC_50_ values of DPPH and ABTS radicals scavenged by CFLs extracts presented extremely significant correlations to TPC (*p* < 0.001) with Pearson’s correlation coefficients of −0.950 and −0.949, respectively. Meanwhile, the Pearson’s correlation coefficient between the EC_50_ of hydroxyl radical and PC was −0.938, implying an extremely significant positive correlation between the hydroxyl radical clearing ability and PC. The reducing power tended to increase with TPC, with a correlation coefficient of 0.972 (*p* < 0.001), suggesting an extremely significant positive correlation. Moreover, the EC_50_ of hydroxyl radical presented a highly significant correlation (*p* < 0.01) to TPC with a Pearson’s correlation coefficient of −0.888. Rocchetti et al. (2020) confirmed the correlation between phenolic compounds and the scavenging ability for DPPH and ABTS radicals using Pearson’s correlation analysis. In this study, highly significant correlations were observed between PC and EC_50_ of DPPH and ABTS radicals with Pearson’s correlation coefficients of −0.854 (*p* < 0.01) and −0.836 (*p* < 0.01), respectively. The Pearson’s correlation coefficient between the EC_50_ of HCCLM6 cell toxicity and TSC was −0.813 (*p* < 0.01), indicating a highly significant correlation between both factors.

## 4 Discussion

The initial separation of bioactive components can be performed according to their distribution coefficient in polar solvents. Accordingly, as one of commonly applied polar solvents for most bioactive substances (Paula et al., 2003), methanol was found suitable for extracting the main active substances such as flavonoids, polyphenols, polysaccharides, and saponins from CFLs in this work.

The total methanol extract of CFLs was then performed as sequential extraction, affording PEE, EAE, and WE. Among them, EAE had the highest TPC of 164.18 ± 12.05 mg GAE/g, WE had the maximum PC content of 373.27 ± 8.67 mg GE/g, and PEE had the largest TSC of 197.35 ± 16.21 mg OAE/g. Plant extracts are known to possess antioxidant properties (Kalidindi et al., 2015; Nurcholis et al., 2021; Saravanakumar et al., 2021) that can be exploited to control the damage exerted by the strong oxidation effect of free radicals, which are atoms, molecules, ions, or groups bearing an unpaired electron produced by enzymatic reactions in human metabolism. Studies have shown that mushroom polysaccharides exhibited strong scavenging ability against ABTS and hydroxyl radicals in a dose-dependent manner (Li et al., 2012). In addition, Rusu et al. (2019) have found that the water-acetone extract of hazelnut (*Corylus avellana* L.) showed significant *in vitro* antioxidant capacity due to its richness in 11 polyphenols including epicatechin, catechin, syringic acid, gallic acid, and protocatechuic acid, *etc.* Wang et al. (2019) have discovered that the ethanol-water extracts from *Olea europaea* leaves contained phenolic compounds, such as luteolin and apigenin, which exhibited dose-dependent scavenging activities against DPPH and superoxide radicals. Here, the three CFLs extracts were assessed for their *in vitro* antioxidant activities. EAE exhibited the strongest antioxidant capacity, which was consistent with the study on the antioxidant capacity of *Ilex latifolia* Thunb extracts (Hu et al., 2014). This may be attributed to the high solubility of polyphenols in ethyl acetate. Khanam et al. (2015) found that polyphenols were present in the ethyl acetate extract of *Eurycoma longifolia* but not in the petroleum ether extract. Moreover, Hassan et al. (2015) revealed that the ethyl acetate extracts of *E. longifolia* contained high content in polyphenols and exhibited strong antioxidant ability.

Natural medicine, especially Chinese medicine, plays a critical role in the treatment of cancer (Song et al., 2018; Zhang et al., 2018). Plant leaves have been proven to be an important source of natural antitumor drugs. For instance, aqueous extracts of *Carica papaya* leaves were found to inhibit the viability of K562 tumor cells (Otsuki et al., 2010). Al-Nemari et al. (2022) demonstrated that the leave extracts of *Annona squamosa* could effectively inhibit MCF-7 and MDA-MB-231 tumor cells. Moreover, acidic polysaccharides from *Gynostemma pentaphyllum* have been found to possess significant inhibitory activities against SPC-A-1 and MGC-803 cells (Yu et al., 2020). He et al. (2021) also found that *Panax quinquefolius* saponins possessed an inhibitory effect on DU145 cell viability. The present study demonstrates that the cell viability of HCCLM6 and HGC27 was significantly decreased after being treated with WE, which may be due to its high PC (Saidan et al., 2015). In fact, the antitumor activity of plant polysaccharides is one of their most prominent features (Zhang et al., 2007). Luo et al. (2014) revealed that water extracts of *Coriolus versicolor* had obvious inhibitory properties against breast cancer cells 4T1. In this study, we found that CFLs extracts inhibited cancer cell viability by promoting HCCLM6 and HGC27 cells early apoptosis. Similarly, Se-POP-3 has been shown to inhibit the proliferation of HepG2 and MCF-7 cells by promoting their early apoptosis (Zhang et al., 2020).

## 5 Conclusion

In the present work, significant deviations were observed in the active substances content of CFLs extracts obtained *via* sequential extraction, with EAE containing the highest TPC and WE and PEE having the maximum PC and TSC, respectively. PEE, EAE, and WE exhibited significant *in vitro* antioxidant capacity, with EAE showing the strongest scavenging ability for DPPH and ABTS radicals and the highest reducing power. Moreover, all three extracts especially WE, could remarkably inhibit the viability of HCCLM6 and HGC27 cells. Finally, the three extracts suppressed the growth of HCCLM6 and HGC27 cells *via* promoting the early apoptosis. Based on the results of this work, EAE and WE should be subjected to further purification to obtain high-purity compounds and to investigate their potential use in the treatment of free radical-related diseases. Moreover, the potential of WE as an antitumor drug merits further investigation. This work provides a reference for the utilization of *C. fascicularis* as a natural medicine.

## Data Availability

The original contributions presented in the study are included in the article/supplementary material, further inquiries can be directed to the corresponding authors.
